# Patterns and Trends in Accidental Poisoning Deaths: Pennsylvania’s Experience 1979-2014

**DOI:** 10.1371/journal.pone.0151655

**Published:** 2016-03-10

**Authors:** Lauren C. Balmert, Jeanine M. Buchanich, Janice L. Pringle, Karl E. Williams, Donald S. Burke, Gary M. Marsh

**Affiliations:** 1 Department of Biostatistics, Graduate School of Public Health, University of Pittsburgh, 130 DeSoto Street, Pittsburgh, PA 15261, United States of America; 2 School of Pharmacy, University of Pittsburgh, 5607 Baum Boulevard, Room 531, Pittsburgh, PA 15206, United States of America; 3 Office of the Medical Examiner of Allegheny County, 1520 Penn Avenue, Pittsburgh, PA 15222, United States of America; 4 Graduate School of Public Health, University of Pittsburgh, 130 DeSoto Street, Pittsburgh, PA 15261, United States of America; Centers for Disease Control and Prevention, UNITED STATES

## Abstract

**Introduction:**

The purpose of this study was to examine county and state-level accidental poisoning mortality trends in Pennsylvania from 1979 to 2014.

**Methods:**

Crude and age-adjusted death rates were formed for age group, race, sex, and county for accidental poisonings (ICD 10 codes X40-X49) from 1979 to 2014 for ages 15+ using the Mortality and Population Data System housed at the University of Pittsburgh. Rate ratios were calculated comparing rates from 1979 to 2014, overall and by sex, age group, and race. Joinpoint regression was used to detect statistically significant changes in trends of age-adjusted mortality rates.

**Results:**

Rate ratios for accidental poisoning mortality in Pennsylvania increased more than 14-fold from 1979 to 2014. The largest rate ratios were among 35–44 year olds, females, and White adults. The highest accidental poisoning mortality rates were found in the counties of Southwestern Pennsylvania, those surrounding Philadelphia, and those in Northeast Pennsylvania near Scranton.

**Conclusions:**

The patterns and locations of accidental poisoning mortality by race, sex, and age group provide direction for interventions and policy makers. In particular, this study found the highest rate ratios in PA among females, whites, and the age group 35–44.

## Introduction

The US currently averages 110 legal and illegal drug overdose deaths daily [1]. Deaths from accidental poisonings, or overdose, have been increasing in the US in recent decades at alarming rates [2, 3]. In 2008, accidental poisonings overtook motor vehicle accidents as the leading cause of accidental death in the US, with more than 41,000 deaths [2]. Analyses of US accidental poisoning deaths have found higher rates in certain areas, including Pennsylvania (PA) [2, 4]. In fact, Pennsylvania was one of 30 states in 2008 with poisoning as the leading cause of accidental death, and one of 20 states with a statistically significantly higher rate compared to the US average [2].

The purpose of this study was to examine county and state-level accidental poisoning mortality trends in PA from 1979 to 2014. These analyses will identify specific demographic and geographic subgroups most at risk for accidental poisoning death to help inform physicians, law enforcement, and public health decision makers. County level analysis will help target specific areas of the state for which to direct resources and interventions.

## Methods

Death counts and rates were compiled using the Mortality and Population Data System (MPDS), which is a repository and retrieval system for detailed mortality data obtained from the National Center for Health Statistics (NCHS) and the US Census [5]. The NCHS provided death counts from electronic death certificate records of each state according to the University of Pittsburgh Department of Biostatistics as part of a Data Use Agreement. The MPDS database contains death counts and populations for the entire US and at the state and county level by race, sex, and age group. Death counts were linked within MPDS to the corresponding population data from the US Census to examine annual counts and death rates for age groups (15–24, 25–34, 35-44-45-54, 55–64, 65–74, 75–84, and 85+), race (white, black, other), sex, and PA county for accidental drug poisonings from 1979 to 2014 (S1 Dataset, S2 Dataset). We excluded analysis of deaths in age groups younger than 15 due to small numbers. We included only deaths from Accidental drug poisoning, which are those with ICD codes E850-E869 (9th revision) and X40-X49 (10th revision), as indicated on individual death certificates. The underlying cause of death code is created from the underlying cause of death text using an NCHS-specific algorithm. A coroner/medical examiner determines the cause of death in accidental poisoning cases; the text comes from that report.

Analyses were limited to the time period corresponding to ICD 9^th^ and 10^th^ revision codes as changes in accidental poisoning coding made comparisons to data before 1979 impossible. To account for changes in coding between the 9^th^ and 10^th^ revisions, the comparability ratio of 1.195 was applied to 9^th^ revision data [6]. Age-adjusted mortality rates were calculated using the 2000 standard million weights. There were no known missing deaths, and there were no deaths missing gender or race. There were fewer than 10 deaths (1999–2014) in the entire state missing age group, and these deaths were excluded from the analyses.

Rate ratios (RR) and corresponding 95% confidence intervals (CI) were calculated comparing rates from 1979–2014, overall and by sex, age group, and race. Confidence intervals for rate ratios were calculated via the epitab csi command in STATA, which provides rate ratios and corresponding 95% confidence intervals [7]. Standard errors for the county level and state level rates were calculated as:

$\mathrm{Standard error of death rate}=\mathrm{death rate}*\left(\frac{\mathrm{Relative standard error of death rate}}{100}\right)$, where the relative standard error of death rate is $100*\sqrt{\frac{1}{Deaths}}$. Confidence intervals were calculated as the death rate plus or minus 1.96 times the standard error of the death rate. Confidence intervals for small samples and for rates prior to 1999 with comparability ratios were calculated via formulas detailed by the CDC [8]. Statistically significant differences in rates and rate ratios of subgroups were indicated by non-overlapping confidence intervals. Joinpoint regression was used to detect statistically significant changes in trends of age-adjusted mortality rates overall, and by sex and race and to detect statistically significant changes in trends of rates for age-specific categories. Average annual percent change (AAPC) was calculated for each model as a weighted average of slope coefficients from the joinpoint regression line, for use in comparing across models with different numbers of joinpoints [9]. Specifically, annual percent change (APC) was calculated as (exp(*β* − 1) * 100 for each significant interval identified by the joinpoint model, where β is the estimated slope coefficient. The AAPC was then calculated as ($\text{exp}\;\left(\frac{\sum w_{i}\beta_{i}}{\sum w_{i}}\right)$), with weights (w_i_) equal to the length of each interval.

Rates were examined graphically by sex and age group over time (1979–2014) and by race-sex group for the 2010–2014 time period. County-level rates were also investigated by race, sex, and age group for 2010 to 2014. Rates were combined for the 5 year period due to small sample sizes and to provide more reliable estimates. We suppressed data if there were fewer than 10 deaths in the cell per NCHS guidelines [10]. Our analyses focus on identifying differences in patterns among the various demographic groups examined. Because all subjects in this analysis were deceased, it was exempt from University of Pittsburgh Institutional Review Board review.

## Results

As shown in Table 1, RRs for accidental poisoning mortality in PA increased more than 14-fold comparing 1979 to 2014. The RR was higher in females than in males, and in whites compared to blacks. The 35–44 year old age group had the highest RR of any considered with almost a 22-fold increase in the time period examined. This age group also had the highest AAPC. However, the highest rate of death in 2014 was among 25–34 year olds (40/100,000).

**Table 1:** Counts and crude rates per 100,000 of accidental poisoning deaths among persons age 15 to 64, by selected characteristics.

	1979^2^			2014						
	Deaths	Rate	95% CI	Deaths	Rate	95% CI	Rate Ratio	95% CI	AAPC^1^	95% CI
**Overall**	134	2.04	(1.7, 2.4)	2458	29.16	(28.0, 30.3)	14.29	(12.0, 17.0)	8.3	(6.5, 10.2)
**Sex**										
Male	96	3.02	(2.4, 3.7)	1633	38.89	(37.0, 40.8)	12.88	(10.5, 15.8)	7.9	(6.0, 9.8)
Female	38	1.12	(0.8, 1.6)	825	19.50	(18.2, 20.8)	17.41	(12.6, 24.1)	9.4	(9.0, 9.9)
**Age Group**										
15–24	43	2.38	(1.7, 3.2)	277	16.25	(14.3, 18.2)	6.83	(5.0, 9.4)	6.0	(4.1, 8.0)
25–34	30	2.04	(1.3, 3.0)	654	39.87	(36.8, 42.9)	19.54	(13.6, 28.2)	8.5	(7.0, 10.0)
35–44	18	1.70	(1.0, 2.7)	555	36.69	(33.6, 39.7)	21.60	(13.5, 34.5)	9.5	(7.8, 11.2)
45–54	24	2.17	(1.6, 2.3)	616	34.10	(31.4, 36.8)	15.71	(10.5, 23.6)	8.2	(4.3, 12.2)
55–64	19	1.70	(1.6, 4.7)	356	20.10	(18.1, 22,2)	11. 82	(7.5, 18.8)	7.8	(6.4, 9.2)
**Race**										
White	118	1.98	(1.6, 2.3)	2160	30.73	(29.4, 32.0)	15.52	(12.9, 18.7)	8.9	(6.8, 10.9)
Black	16	2.85	(1.6, 4.7)	283	27.00	(23.9, 30.2)	9.47	(5.7, 15.7)	5.0	(3.2, 6.8)
Other	0	0.00	—	15	4.24	(2.4, 7.0)	—	—	—	

^1^AAPC from joinpoint regression using age-adjusted mortality rates

^2^Using comparability ratio of 1.195

**Fig 1:**
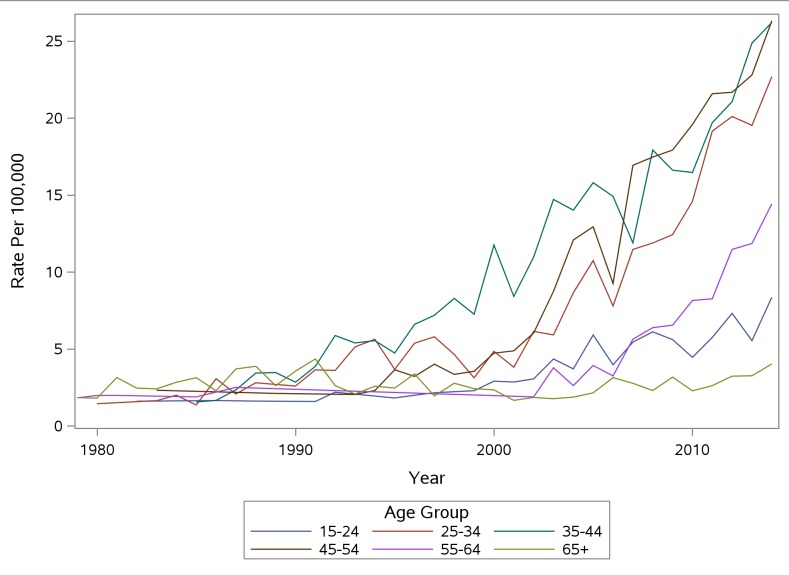
PA Accidental Poisoning Mortality Rate Per 100,000 Males by Age Group.

**Fig 2:**
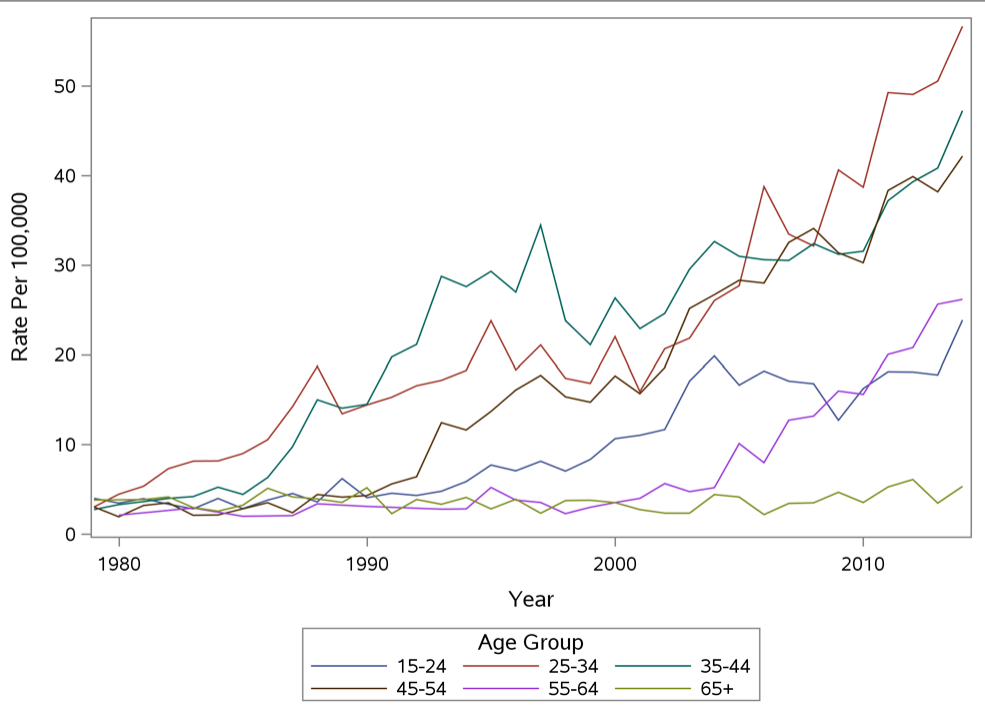
PA Accidental Poisoning Mortality Rate Per 100,000 Females by Age Group.

Mortality rates among males increased for most age groups from 1979 to 2014 (Fig 1). Rate increases occurred the earliest for 25–34 year olds, followed over by time by 35–44 year olds, then 45–54 years old. Rates did not begin to increase among 15–24 years olds until the late 1990s. Beginning in 2009, rates for males age 25–34 increased dramatically and in 2014 were the highest among any age group. Rates for ages 65+ have remained relatively stable over the time period examined. Rates for females showed similar patterns, although the highest rates identified were approximately 26/100,000 in 2014 among 35–44 year olds and 45–54 year olds compared to 57/100,000 for 25–34 year old males in 2014 (Fig 2). Increases for females occurred more recently than for males, where increases began in the late 1980s.

**Fig 3:**
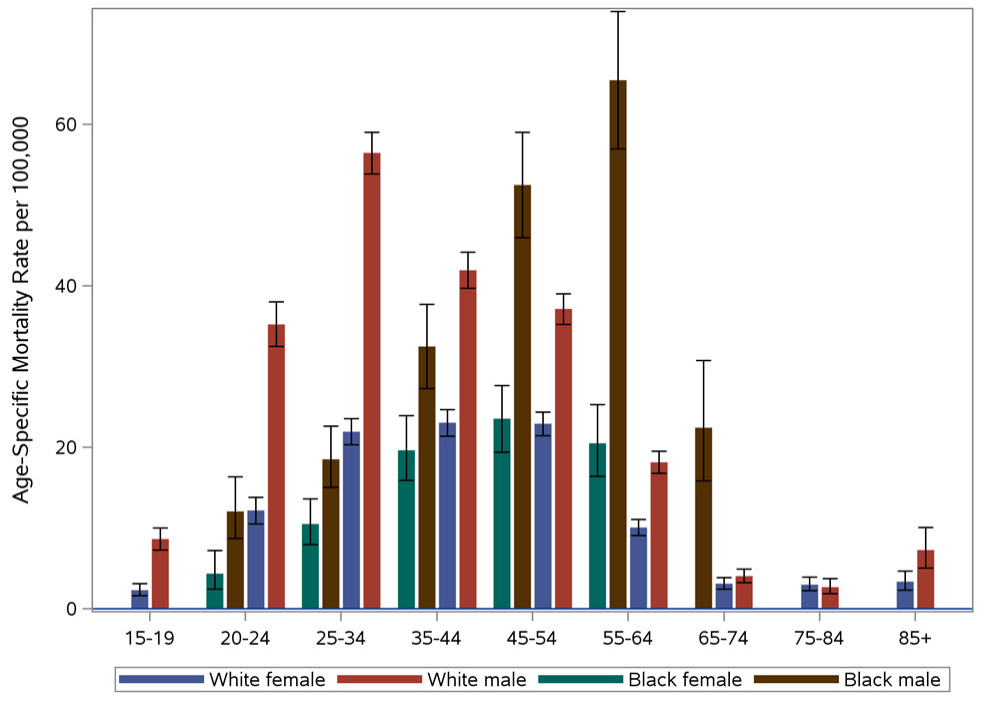
PA Age-Specific Accidental Poisoning Mortality Rates Per 100,000 by Race and Sex, 2010–2014.

Fig 3 shows the race-sex specific mortality rates by age-group for the 2010–2014 time period (rates based on fewer than 10 deaths have been suppressed). As seen, white male mortality rates peak at 25–34 years old. Rates among black males increase steadily to peak at 55–64 years old. The rates for white and black females are more similar than those for males, with both peaking at ages 45–54.

**Fig 4:**
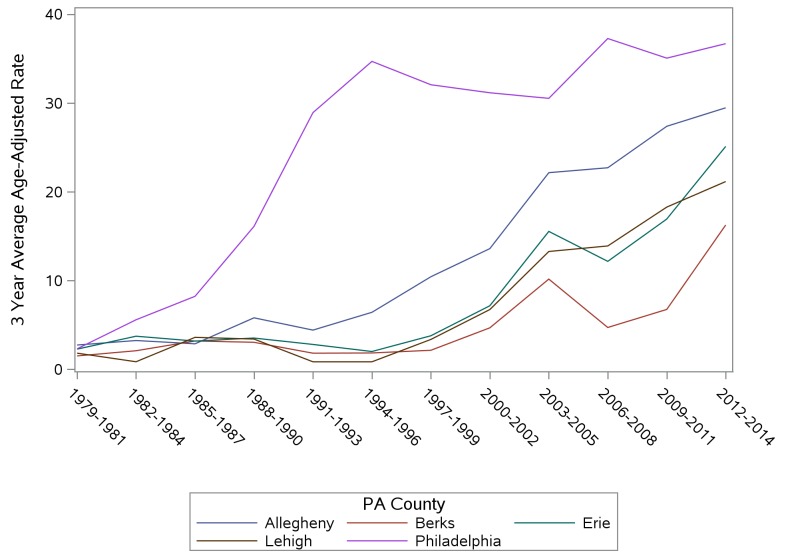
Three Year Average Age-Adjusted Poisoning Mortality Rates for Counties with the Top 5 Largest PA Cities, 1979–2014.

Examinations of mortality patterns by county indicated the highest rates for males (2010–2014) in the counties of Southwestern PA, those surrounding Philadelphia, and those in Northeast PA near Scranton (data not shown). High rates for females were found in counties surrounding Pittsburgh in Southwestern PA, in Philadelphia County and those counties in Northeastern PA (data not shown). Fig 4 shows three year average age-adjusted mortality rates (1979–2014) for the PA counties with the five largest cities (Allegheny: Pittsburgh; Berks: Reading; Erie: Erie; Lehigh: Allentown; Philadelphia: Philadelphia). Accidental poisoning death rates began increasing in Philadelphia County in the early 1980s and increase from less than 5/100,000 in 1980 to greater than 35/100,000 in 1992. They have been the highest of any county since 1982. Rates in Allegheny County began increasing rapidly in the mid 1990s and were nearly as high as Philadelphia County by 2013. Rates in the other three counties began increasing later, although rates in Erie County have seen rapid increases since 2007.

The PA county with the highest 2010–2014 mortality rate for each race-sex-age group is shown in Table 2. Philadelphia County has the highest rates for male and female blacks until 35–44 years old; after age 45, Allegheny County has the highest rates for black males and females. The highest areas for young white males and females are suburban or rural PA counties. Bucks County, a suburb of Philadelphia, and Carbon County, in northeastern PA, have the highest rates for 20–24 year old white females and males, respectively. More rural Washington and Armstrong Counties have the highest mortality rates for white females ages 25–34 and 35–44, respectively. Cambria County, in which Johnstown is located, has the highest mortality rate for white females ages 45–54. Philadelphia County has the highest mortality rates for white males and females in the oldest age group examined and the second highest mortality rate for any race sex age group examined (121/100,000).

**Table 2:** PA County with Highest 2010–2014 Accidental Poisoning Mortality Rate by Race, Sex, and Age Group.

	20–24 Years	25–34 Years	35–44 Years	45–54 Years	55–64 Years
	County (Rate)	County (Rate)	County (Rate)	County (Rate)	County (Rate)
	(95% CI)	(95% CI)	(95% CI)	(95% CI)	(95% CI)
**Black**					
Female	Suppressed	Philadelphia (12.2)	Philadelphia (25.8)	Allegheny (36.7)	Allegheny (33.7)
		(8.4, 17.2)	(19.7, 33.1)	(23.0, 55.6)	(20.0, 53.3)
Male	Philadelphia (13.7)	Philadelphia (24.9)	Philadelphia (48.7)	Allegheny (85.0)	Allegheny (94.3)
	(8.4, 21.1)	(18.7, 32.5)	(39.2, 59.8)	(61.0, 115.4)	(67.3, 128.4)
**White**					
Female	Bucks (21.7)	Washington (37.9)	Armstrong (68.8)	Cambria (60.1)	Philadelphia (21.6)
	(12.4, 35.4)	(22.8, 59.1)	(37.6, 115.4)	(40.3, 86.3)	(15.8, 28.8)
Male	Carbon (126.7)	Susquehanna (113.3)	Philadelphia (97.1)	Philadelphia (121.4)	Philadelphia (55.5)
	(60.7, 232.9)	(58.6, 198.0)	(12.2, 37.6)	(106.7, 136.1)	(45.1, 66.0)

## Discussion

This analysis is the first to examine in detail PA accidental poisoning mortality over time. Accidental overdose mortality rates have been found to be significantly higher in PA compared to the US average [2, 4]. These results warranted further investigation of demographic factors at the state and county level, as the patterns of accidental poisoning mortality by race, sex, and age group provide important information for targeting interventions in PA.

While this analysis examined accidental poisoning deaths in Pennsylvania, many of these findings are applicable to other states as well. Male accidental poisoning mortality rates remain higher than females; however, females saw a more dramatic increase in rates from 1979 to 2014. For both males and females, the age groups 25–54 have considerably higher rates over the last decade. Among white males in PA, overdose occurred most frequently in 25–44 year olds, but among black males, overdose occurred most frequently in 45–65 year olds. We speculate that these differences in age groups reflect different patterns of drug usage among blacks and whites. The National Survey on Drug Use and Health 2012 report indicates higher prevalence of cocaine, hallucinogen, inhalant, and non-medical pain-killer use among white adults (26+) and a higher prevalence of crack cocaine use among black adults (26+) [11]. Additionally, a study of heroin use among patients entering substance abuse treatment centers indicates a shift to predominately white users in the last 50 years [12].

In the US, drug overdose death rates peak at ages 45–54, and rates are considerably lower at ages 35–44 (and earlier) and 55–64 (and later) [13]. In this examination of PA death rates, rates among white females remained relatively stable from ages 25–54, and remained stable among black females from ages 35–64, before declining. These findings seem to indicate a more prolonged period of concern for overdoses in PA women compared to the US. This could also indicate that there are areas where women are at an increased risk for accidental poisoning death that would only be found by examining state and county patterns.

Women are more prone to having accelerated progression from time of first use to substance abuse, a phenomenon described by Greenfield et al., as “telescoping” [14], and often enter treatment programs with more severe dependence than men who have used for a comparable period of time [15]. Additionally, most women who enter substance abuse treatment programs have other factors affecting their rehabilitation including being responsible for children [14, 16], and being more reliant on public insurance [17]. These factors could potentially affect a woman’s decision to enter or remain in a drug rehabilitation program. While women and men are equally likely to be successful in drug treatment programs [14], these women’s family considerations and reliance on public support indicate that multiple agencies and departments must work together to treat women with substance abuse problems holistically.

The county level findings provide possible avenues for targeting interventions to areas with the highest mortality from accidental poisoning. Counties with the highest 2010–2014 rates in females are primarily in suburban southwest PA. In males, the highest prevalence rates are more widespread and include both southwest and southeast PA, plus the northeast area including Carbon and Susquehanna. These patterns emphasize that, currently, accidental poisoning deaths especially among white females are occurring in suburban and rural areas. Other area-specific analyses should focus on non-urban mortality patterns.

Examination of rates for the counties with the largest PA cities indicates that areas such as Erie County and Lehigh County have seen rapid increases in accidental poisoning deaths since 2010. These are not large urban areas, such as Pittsburgh or Philadelphia, but rather counties with urbanized areas of more than 50,000 people [18]. Public and mental health agencies and law enforcement in those areas may need additional resources as death rates approach those found in the much larger urban areas, such as those seen in Pittsburgh and Philadelphia, PA.

Because of data limitations only data through 2014 could be examined. However, media reports indicate the overdose problem in PA and in the US is continuing to grow. This growth was recently evident in Washington County, PA between August 17 and 18, 2015 when 25 people overdosed on heroin laced with fentanyl, 3 of them fatally [1]. Similar outbreaks of overdoses have recently occurred in urban areas, like Chicago, and semi-rural areas, like Nash County, NC. A better understanding of the demographic characteristics of these deaths will assist law enforcement and public health officials in appropriately targeting resources like naloxone [19, 20].

PA is making efforts to combat overdoses. In September 2014, Act 139 of 2014 was signed into law allowing first responders to carry naloxone for reversing overdoses [21]. As of September 1, 2015, 302 overdoses had been reversed using naloxone [22], including three in Washington County in August 2015. More than 2/3 of the others were in Philadelphia County and its immediate surrounding counties. Additionally, the CDC recently granted the PA Department of Health funding to battle the epidemic of drug overdoses, particularly from prescription opioid use [23], by focusing on strengthening the Prescription Drug Monitoring Program. Forty-one states and the District of Columbia now have laws allowing access to naloxone, and 30 states have laws providing immunity from criminal prosecution to those who prescribe, dispense, or distribute it [24].

This study was subject to several limitations. First, these analyses focused on accidental poisonings as the underlying cause of death (codes E860-E869 and X40-X49), excluding deaths where the intent was unknown and where poisoning may have been a contributory, and not the underlying, cause of death. Cause of death determination in these cases is made by a coroner or medical examiner. Because in PA these are county-specific positions, there could be reporting bias if a coroner classified deaths as unknown rather than accidental. We examined county-specific proportion of deaths classified as unknown and accidental and found little variation by county with the exception of Berks, PA which had a higher proportion of unknown. If this was a systematic reporting error, there could be more accidental poisoning deaths in Berks County than reported using these data. Second, death certificates may not accurately capture accidental poisonings due to specific drugs, such as heroin or opioids, so those analyses were precluded by the data [25]. Given the lack of standardized assessment and reporting methods for classifying accidental poisoning death based on toxicology screens, there may be substantial variation in how coroners and medical examiners perform and report toxicology screens. Third, analyses were limited to ICD-9 and ICD-10 (1979 and onward) due to changes in underlying cause of death coding which made comparisons with earlier versions impossible. Additionally, data were suppressed if counts or rates were based on fewer than 10 deaths, due to NCHS reporting restrictions. Only PA counties with higher populations of blacks could have or would have been included in analyses examining rates by race. Therefore, it is not surprising that Allegheny and Philadelphia Counties were the only ones represented in Table 2 when examining the highest rates among blacks in PA.

## Conclusions

This study identified patterns and geographic locations of accidental poisoning mortality by race, sex, and age group. Substantial increases in rates from 1979 to 2014 were found particularly among females, whites, and those in the age group 35–44. Public and mental health officials should be mindful of the unique patterns of accidental poisoning death in the Commonwealth, including among young whites and older blacks. Interventions to reduce overdoses in PA should be designed and implemented to appropriately target these demographics.

## Supporting Information

S1 Dataset Pennsylvania Accidental Poisoning Mortality Data. (XLSX)

S2 Dataset Pennsylvania County Accidental Poisoning Mortality Data. (XLSX)
