# Correction: Patterns and Trends in Accidental Poisoning Deaths: Pennsylvania's Experience 1979-2014

**DOI:** 10.1371/journal.pone.0159469

**Published:** 2016-07-13

**Authors:** Lauren C. Balmert, Jeanine M. Buchanich, Janice L. Pringle, Karl E. Williams, Donald S. Burke, Gary M. Marsh

The images for Figs 1 and 2 are incorrectly switched. The image that appears as Fig 1 should be Fig 2, and the image that appears as Fig 2 should be Fig 1. The figure captions appear in the correct order. Please see the corrected Fig 1 and Fig 2 here.

**Fig 1:**
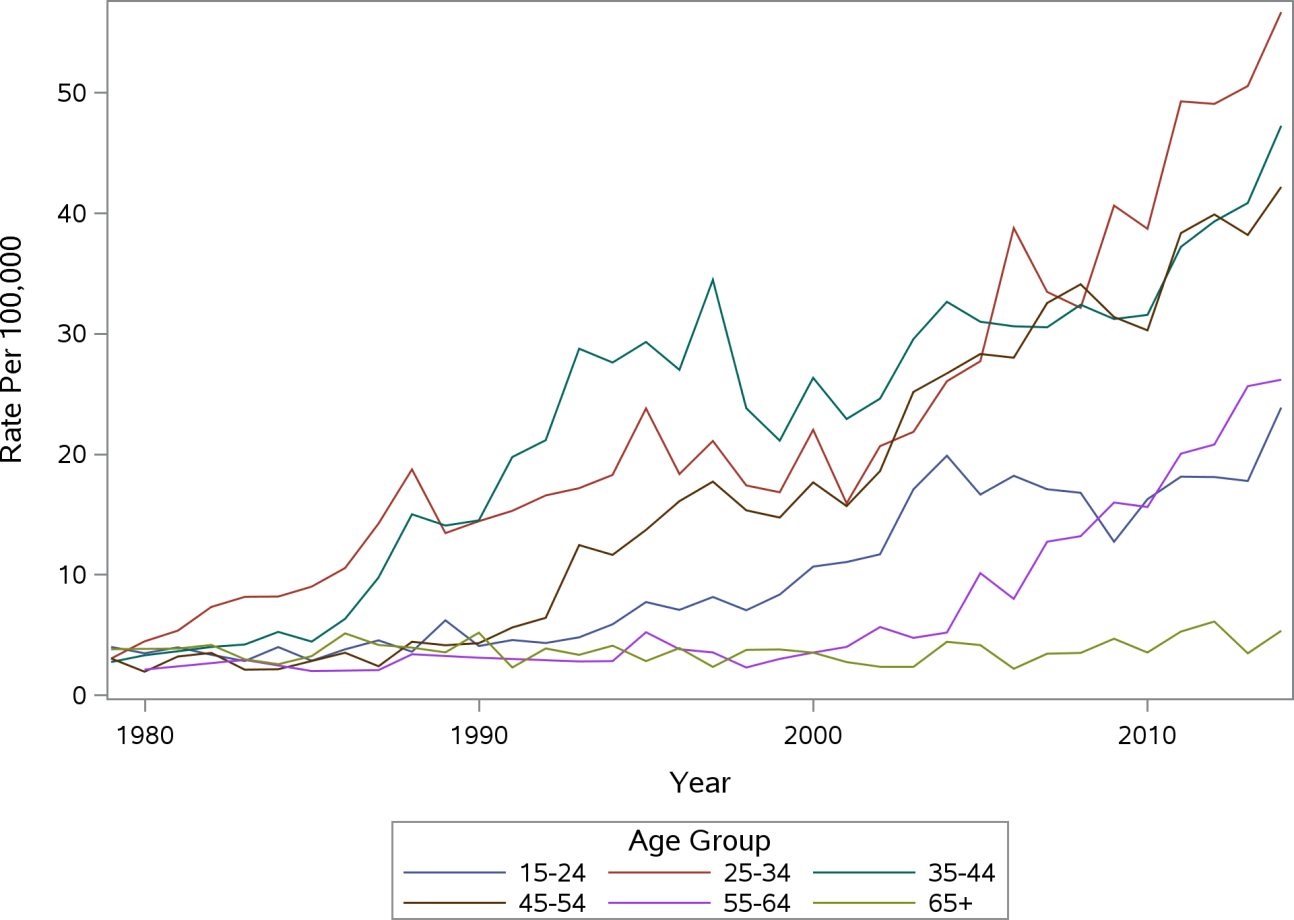
PA Accidental Poisoning Mortality Rate Per 100,000 Males by Age Group.

**Fig 2:**
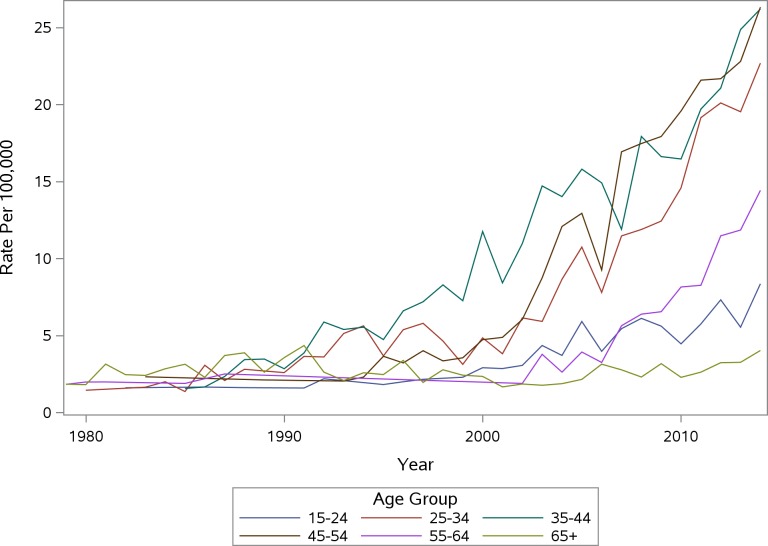
PA Accidental Poisoning Mortality Rate Per 100,000 Females by Age Group.
